# Milk Protein Hydrolysis by Actinidin—Kinetic and Thermodynamic Characterisation and Comparison to Bromelain and Papain

**DOI:** 10.3390/foods12234248

**Published:** 2023-11-24

**Authors:** Surjit Kaur, Todor Vasiljevic, Thom Huppertz

**Affiliations:** 1 Advanced Food Systems Research Unit, Institute for Sustainable Industries & Liveable Cities, College of Health and Biomedicine, Victoria University, Melbourne, VIC 8001, Australia; surjit.kaur@live.vu.edu.au (S.K.); todor.vasiljevic@vu.edu.au (T.V.); 2 FrieslandCampina, 3818 LE Amersfoort, The Netherlands; 3 Food Quality & Design Group, Wageningen University & Research, 6708 WG Wageningen, The Netherlands

**Keywords:** actinidin, papain, bromelain, proteolysis, milk protein concentrate, whey protein concentrate, whey protein isolate, kinetics, thermodynamic parameters

## Abstract

Plant proteases, including actinidin, papain and bromelain, have been widely used in the food industry but with limited application in dairy systems. This research aimed to establish and compare operational parameters (kinetics, temperature, enzyme type, time and thermodynamics) relevant to the applications of these enzymes in the hydrolysis of whey protein isolates (WPI), whey protein concentrates (WPC) or milk protein concentrates (MPC). The degree of hydrolysis (DH) increased with the rise in temperature, and the maximum DH was achieved at 60 °C for all three dairy systems. The addition of papain resulted in a greater %DH of whey proteins in comparison to bromelain. The cleavage of proteins was clearly time-dependent (*p* < 0.05), while the pH did not change significantly (*p* > 0.05) during this time. PAGE analysis revealed that all three enzymes mainly acted on α-lactalbumin and α_s_-casein in WPI and MPC, respectively. Kinetic parameters from the Lineweaver–Burk plot at 60 °C using WPC and MPC as a substrate varied widely, establishing that WPC hydrolysis was characterised by a lower KM, higher kcat, kcat/KM and Vmax compared to MPC in the case of all three enzymes. The difference in kcat/KM values amongst all enzymes (actinidin > papain > bromelain) indicated the difference in the strength of substrate binding sites. The thermodynamic parameters of these enzymes with MPC and WPC were also determined at a temperature range of 15–60 °C, and the results indicate the potential application of papain and actinidin in the dairy industry.

## 1. Introduction

Cysteine proteases (EC.3.4.22), such as actinidin (EC 3.4.22.14), bromelain (EC 3.4.22.32) and papain (EC 3.4.22.2), are plant-based enzymes with a similar substrate specificity, a tertiary structure and amino acid chains [1]. Actinidin contains 220 amino acids and has a molecular weight of 23.5 kDa, bromelain has 285 amino acids and a molecular weight range of 23.4–35.7 kDa and papain has 212 amino acids with a 23.4 kDa molecular weight [1]. These proteases have several advantages, such as mild processing conditions, faster processing rates and economic, health and safety benefits that enable industries to reduce production costs and improve efficiency/productivity [2]. Furthermore, they are also active over a wide range of temperatures and pH. For example, actinidin is active in the pH range 4-10 and between 15 and 60 °C [3,4], bromelain is active at pH 4.6-9.5 and at 10–75 °C [5], and papain is active in the narrower pH range of 5–7 but still remains active at higher temperatures (<90 °C) [2]. Despite having all these common advantages, these enzymes may behave differently even under the same processing conditions; their thorough kinetic and thermodynamic characterisation is needed to enhance knowledge in this field. 

Various studies have shown that these proteases enhance gastric digestion [6], while their impact on the intestinal phase of digestion is rather minor but still noticeable [7,8]. They have been extensively used as meat tenderisers due to their proteolytic actions on collagen and myofibrillar proteins [9,10,11], as well as beer clarifiers and milk-coagulating agents [12,13]. The proteolytic activity of actinidin has also been used in some dairy systems to minimise antigenicity (allergenicity) and improve functionalities [6,14,15,16]. Comparatively, however, there is still limited information available for commercial applications of actinidin, bromelain and papain in these dairy systems. 

Milk proteins (MP) are widely used in various food products. However, the application of proteins in food systems is sometimes limited due to issues related to allergenicity [17], digestibility [11], solubility or heat stability [18,19]. In some of these cases, the use of protein hydrolysates rather than intact proteins can provide viable solutions. Therefore, the efficient utilisation of milk proteins in food systems depends on tailoring their structural characteristics. Establishing optimal conditions for protein hydrolysis is crucial to obtaining protein hydrolysates with improved structural characteristics [20].

In our previous work, actinidin was used to determine the potential mode of its action and its potential benefits with dairy systems [14,15,21], and this work showed the appreciable effect of actinidin with dairy systems, especially when minimising antigenicity and improving functionalities [14,15]. Therefore, these positive outcomes lead to further exploration of its applications on an industrial basis to develop noble products and processes. To consider industrial applications, however, the development of the process needs to be considered. For this, and to enable adequate process control, the kinetics of hydrolysis reactions are required. Hence, the aim of the current study was to determine the kinetic and thermodynamic parameters of the actinidin-induced hydrolysis of milk proteins. Our second aim was to compare such kinetic and thermodynamic parameters for actinidin with those for the two other aforementioned cysteine proteases, i.e., papain and bromelain. For this purpose, the papain and bromelain-induced hydrolysis of the main proteins in the whey protein isolate (WPI), whey protein concentrate (WPC) and milk protein concentrate (MPC) were studied, and data among those previously reported for actinidin-induced protein hydrolysis in the same ingredients [21] were used to determine appropriate thermodynamic and kinetic parameters. The research question was whether these proteases showed similar behaviour to actinidin as toward the tested milk proteins. The establishment of such a parameter can form an important basis for the design and optimisation of (industrial) processes for the controlled hydrolysis of milk proteins using actinidin, papain or bromelain. 

## 2. Materials and Methods

### 2.1. Materials

MPC (80%, *w*/*w*, protein on dry matter), WPI (90%, *w*/*w*, protein on dry matter) and WPC (80%, *w*/*w*, protein on dry matter) were sourced from Fonterra Cooperative (Palmerston North, New Zealand). Papain (60,000 activity units mg^−1^) and bromelain (2200 activity units g^−1^) were obtained from Connell Bros. Company Australasia (Croydon South, Victoria, Australia). Actinidin (KEP500; 521 activity units g^−1^) was obtained from kiwiEnzyme.com Ltd. (Martinborough, Wellington, New Zealand). A total of 0.2125 M of sodium phosphate buffer (pH 8.2) and Trinitrobenzenesulfonic acid (TNBS) were used. Simulated milk ultrafiltrate (SMUF) at pH 6.8 was used as a buffer for all samples, including the controls [21]. All other chemicals were sourced from Sigma-Aldrich Pty Ltd (Castle Hill, NSW, Australia).

### 2.2. Protease-Induced Hydrolysis of Milk Protein Systems

Protein suspensions were prepared at a constant concentration of total solids (5% *w*/*w*) in simulated milk ultrafiltrate (SMUF), as described previously [21]. The protein suspensions were then equilibrated at a specific temperature (15, 40 or 60 °C) before adding each protease. The pH was not controlled but monitored throughout hydrolysis using a portable pH meter (model 3110 SET2 ProfiLine, Xylem Analytics, Hemmant, QLD, Australia). The same level of the proteases was added based on their activity per unit of mass to facilitate the best comparison among them with all three substrates. The proteases were added at the enzyme-to-substrate ratio (E:S) of 1:100 by adding (5.21 units of each enzyme activity g^−1^ of protein) or 1:500 (1.04 units of actinidin g^−1^ of protein; 1:500 was considered for thermodynamic parameters of actinidin only to compare enzyme efficiency). Subsequently, each protease assay was performed at selected temperatures for 5 h with continuous stirring in a water bath (Grant Instruments, Cambridge Ltd., Barrington, Cambridge, England). An aliquot of 1.0 mL of the hydrolysed samples was taken out on an hourly basis, and then 1 mL of 10% (*w*/*v*) SDS was added, followed by heat treatment at 90 °C for 5 min to terminate the reaction. The samples were then stored at −20 °C for further testing. 

### 2.3. Determination of the %DH of Milk Proteins by Papain and Bromelain

As described previously [21], the trinitrobenzenesulfonic acid (TNBS) method [22] was used to determine the number of released peptide bonds by measuring the absorbance of the product formed at a wavelength of 340 nm and using a spectrophotometer (Biochrome Libra S12, Biochrom Ltd., Cambridge, UK). A degree of hydrolysis was calculated on the basis of complete hydrolysis. For this, fully hydrolysed samples were prepared by treating each substrate with 6 M HCl under reflux [23]. In brief, 0.5 g of each sample, with an equivalent protein content to hydrolysates, was obtained via enzymatic hydrolysis, placed in a conical flask with 10 mL of 6M HCl, followed by the addition of 5 to 6 pieces of boiling chips. The test solution was heated at 110 °C in an oil bath in a fume hood under reflux for 24 h. The hydrolysates were then filtered and neutralised, and the absorbance was determined using the TNBS method, as stated previously [21]. A DH was expressed as the percentage of cleaved peptide bonds relative to the completely hydrolysed sample:

(1) $$\%DH=\frac{h}{h-tot}\times 100\;$$

where (*h − tot)* is the total numbers of peptide bonds per protein equivalent obtained from given samples with chemical hydrolysis, and h is the number of hydrolysed bonds.

### 2.4. Kinetic and Thermodynamic Parameters Describing Milk Protein Hydrolysis by All Three Proteases

For the determination of the kinetic parameters, WPC and MPC suspensions were prepared as per Section 2.2 at protein concentrations of 20, 40, 60, 80 or 100 mg mL^−1^. Incubations were performed at 60 °C, and reactions were initiated by adding 2.6 units of the enzyme activity g^−1^ of the actinidin, bromelain or papain protein to subsequent samples. During a period of 60 min of incubation, 1 mL of the aliquots were drawn every 5 min after which the reaction was terminated, as described in Section 2.3. The initial velocity at various enzyme-to-substrate ratios by keeping the enzyme constant and varying the substrate, confirmed that data followed the Michaelis–Menten behaviour for both substrates in all three enzymes. Thus, kinetic parameters were established from the equation generated from a linear trendline of the Lineweaver–Burk plot [24].

(2) $$\frac{1}{V}=\frac{KM}{Vmax\left[s\right]}+\frac{1}{Vmax}$$

where *V* is the enzyme velocity (min^−1^), *S* is the substrate concentration (µg mL^−1^), *KM* is the saturation constant (μg mL^−1^) and *Vmax* is the maximum enzymatic activity for substrate conversion (μg mL^−1^ min^−1^).

For thermodynamic parameters, the hydrolysis process for all enzyme-to-substrate ratios was correlated to the Arrhenius law as follows:

(3) $$k=Ae^{\frac{-Ea}{RT}}$$

where *A* is known as the pre-exponential factor, also called the frequency factor (1/s), *Ea* is the activation energy (kJ mol^−1^), *R* is the molar gas constant (8.314 kJ mol^−1^) and *T* is the treatment temperature in Kelvin. 

So, activation energy was calculated using the following equation:

(4) $$Ea=-RT\ln\left(\frac{k}{A}\right)\;\;$$

The activation energy (*Ea*) was calculated from the slope of the Arrhenius plot, graphed utilising the natural log of the reaction rate constant and inverse of temperature in Kelvin at a temperature range of 15–60 °C. 

### 2.5. Protease-Induced Hydrolysis of Milk Proteins

To monitor the hydrolysis of the individual proteins in WPC and MPC, densitometric analysis was performed on SDS-PAGE gels for samples run under reducing conditions (using β-mercaptoethanol), as described previously [21]. The gels were scanned using an imager (Chemidoc MP, Bio-Rad Laboratories, Hercules, CA, USA) and the band quantifications were performed on all the reducing gels in triplicate via Image Lab software (6.0.1 @2017, Bio-Rad Laboratories Inc., Hercules, CA, USA). 

### 2.6. Statistical Analysis 

All experiments, including hydrolysis and kinetics, were replicated at least three times for all three substrates. The data obtained were analysed as a split-plot design repeated in time measurements with the SAS software (ver. 9.1) and the GLM protocols. Then, the means were compared using the LSMEANS function with the option of PDIFF, and the significance was set at *p* < 0.05. 

## 3. Results

### 3.1. Impact of Processing Conditions on pH and DH 

The change in pH during the proteolysis of MPs with bromelain and papain was monitored throughout the incubation. The decline in pH after 5 h, relative to the controls, is shown in Table 1. From the table, three clear trends can be seen, i.e., (1) in the case of both enzymes, an increase in incubation temperature resulted in a greater decline in the pH; (2) in the case of bromelain, greater a pH decline was observed upon WPC or WPI hydrolysis than that of MPC, particularly at 40 and 60 °C; and (3) in contrast to bromelain, papain activity resulted in a more prominent decrease in pH after the hydrolysis of MPC compared to WPC or WPI and in particular at 60 °C. The greatest decline in pH of 0.44 (bromelain) and 0.35 (papain) was observed at 60 °C for WPI and MPC, respectively (Table 1). The change in pH and %DH for actinidin during the 5h incubation was already reported previously when the maximum pH decline was 0.57 (MPC) and 0.28 (WPI and WPC) at 60 °C [21].

In the current study, the %DH for all samples increased with the increase in the incubation temperature (15 to 60 °C) (Table 2). These results are in line with the previous study in which hydrolysates obtained with actinidin showed the same trend [21]. The %DH of actinidin, obtained under the same conditions, is not included in the current study as it is already reported [21]. Furthermore, both substrate suspensions incubated above 60 °C underwent a sol-gel transition via both bromelain and papain and, thus, could not be analysed further. Notably, %DH did not plateau during the incubation time (5 h). Furthermore, the %DH varied widely between substrates during the determined time and ranged from ∼6 to ∼17%. In the case of the hydrolysates obtained using both bromelain and papain at 60 °C, WPI showed the greatest %DH followed by WPC, whereas MPC showed the lowest %DH (Table 2). Interestingly, whey protein-based ingredients were hydrolysed with papain and reached almost double the %DH as that of bromelain after 5h of incubation at 60 °C.

### 3.2. Proteolysis Patterns for Enzymatic-Induced Hydrolysis of MPC and WPC

The results from monitoring the hydrolysis of individual milk proteins in MPC and WPC via the densitometric analysis of SDS-PAGE gels are shown in Table 3. The patterns of protein hydrolysis were compared and they appeared to be in agreement with the %DH achieved. A substantial reduction in the band intensities was aligned with an increase in the temperature for all samples. Papain showed the greatest band intensity reduction, followed by actinidin and bromelain, respectively. Even the level of MPC hydrolysis with bromelain did not differ much in proportion to the remaining proteins at 15 and 40 °C (Table 3). Of the caseins, the α_s_-CNs appeared more susceptible to hydrolysis than β- or κ-CN, particularly for actinidin and papain (Table 3). As opposed to these observations, bromelain exerted a similar cleavage action on all proteins after 5 h of hydrolysis. For example, for α_s_-, β- or κ-CNs, the proportion of the remaining protein fractions ranged between ~60 and 70% compared to the control. The hydrolytic action of all three enzymes on whey proteins in MPC appeared to be directed at α-LA as β-LG was not affected as much. The trend appeared unchanged with the change in the substrate since actinidin and papain action exerted their activity mainly on α-LA and, to a lesser extent, on β-LG when the WPC was used as a substrate. This was most apparent at 60 °C, with ∼16% and ~9% of β-LG and ∼6% and ~5% of α-LA remaining after 5 h of incubation with actinidin and papain, respectively. Bromelain, however, exerted the low and equal cleavage of these proteins with about 38% of β-LG and ~36% of α-LA remaining (Table 3).

### 3.3. Thermodynamic Characterisation of Actinidin, Bromelain and Papain

The thermodynamic parameters for actinidin, bromelain and papain-induced hydrolysis of milk proteins were established from the Arrhenius plot. Table 4 depicts the dependence of ln *k* with 1/*T* for hydrolysis reactions at various temperatures and incubation times for each protease. In the case of all three enzymes, the Arrhenius plot was fitted with linear functions with a coefficient of correlation ranging from 0.81 to 0.99. At an E:S of 1:100 (5.21 Units g^−1^ of protein), the activation energy of bromelain with MPC was the greatest, whereas papain exhibited the lowest Ea with WPC. The activation energy (Ea) for MPC, WPC and WPI was found to be about 25.4, 23.9 and 24.5 kJ mol^−1^ (for bromelain) and 13.68, 12.07 and 11.05 kJ mol^−1^ (for papain), respectively. Moreover, the activation energy (Ea) for MPC, WPC and WPI with actinidine was found to be 18.1, 15.0 and 14.4 kJ mol^−1^, respectively. When the E:S ratio changed to 1:500 (1.04 Units g^−1^ of protein), the activation energy (Ea) for MPC, WPC and WPI changed to 21.1, 17.1 and 15.3 kJ mol^−1^, respectively (Supplementary Materials, Table S1). The highest activation energy reported was about 25 kJ mol^−1^ in the case of MPC with bromelain, which is far lower than that reported for different proteases and/or substrates. For example, a study with ficin protease reported an activation energy of about 54 kJ mol^−1^ using casein as a substrate [25]. An activation energy of around 74 kJ mol^−1^ was noted for papain using collagen as a substrate [26].

### 3.4. Estimation of Kinetic Parameters for Milk Protein Hydrolysis by Actinidin, Bromelain and Papain at 60 °C

The kinetic parameters, including Vmax, ½Vmax, kcat, KM and kcat/KM were determined for all three enzymes using the Lineweaver–Burk plots and MPC and WPC as the substrates (Table 5). The reciprocal plot of the initial velocity versus substrate concentrations showed very good linearity, providing r^2^ = 0.99 and 0.97 (for actinidin), 0.97 and 0.96 (for bromelain) and 0.96 and 0.93 (for papain) for MPC and WPC, respectively. The Michaelis–Menten constant (KM) was significantly (*p* < 0.05) different between the substrates but showed a consistent trend among the enzymes (papain < actinidin < bromelain).

The lower the KM, the higher the binding affinity of the protease with a particular substrate [27]. In the current study, the lowest KM values were obtained for papain with WPC and MPC at about 1.87 × 10^4^ and 3.05 × 10^4^ μg mL^−1^, respectively. Actinidin had a slightly higher KM with WPC (2.13 × 10^4^ μg mL^−1^) and MPC (3.18 × 10^4^ μg mL^−1^) than papain but was still much lower compared to that of bromelain. Bromelain was characterised as having the highest KM (μg mL^−1^) amongst all the enzymes of 10.71 × 10^4^ and 17.71 × 10^4^ for WPC and MPC, respectively (Table 5). 

With WPC as the substrate, all three proteases were characterised by a low KM value (above stated), a high turnover number (kcat) and high kcat/KM (mL min^−1^ μg^−1^) in comparison to those of MPC (Table 5). These results indicate that all enzymes acted preferentially on the peptide bonds of WPC. Clearly, WPC appears to be a better substrate in comparison to MPC. For example, a turnover number (kcat) of WPC with papain, actinidin and bromelain (0.28, 0.99 and 0.32 min^−1^, respectively) was greater than that of MPC (0.32, 0.39 and 0.25 min^−1^). Also, kcat/KM values for WPC with papain, actinidin and bromelain (15, 46 and 3mL min^−1^ μg^−1^) were much greater than that of MPC (11, 12 and 1 mL min^−1^ μg^−1^) (Table 5).

## 4. Discussion

Operational parameters such as the incubation temperature, pH, substrate type and reaction time are very important variables in order to establish the feasibility of applications of any enzyme, including actinidin, bromelain and papain, in the industry. After obtaining the operational conditions for these proteases with milk protein substrates (Table 1 and Table 2), the thermodynamic (Table 4) and kinetic (Table 5) parameters were determined using WPC and MPC as the substrates to characterise or establish a relevant knowledge base in relation to their enzyme activity. Proteolytic patterns used SDS-PAGE to confirm the degree of hydrolysis of milk proteins (Table 3). 

The current study was conducted without a pH adjustment in order to replicate commercial conditions. The pH reduction took place, but it remained in the range of 6.73–5.99 throughout the process for all enzymes and substrates (Table 1). Overall, the pH change was slight, with papain achieving a greater change in pH at a maximum incubation temperature and time than bromelain, except for WPI. Neutralisation (pH control) usually results in a high salt content, which may the limit implementation of these hydrolysates in certain applications; thus, achieving the required level of hydrolysis without pH control is desirable from an industrial point of view. However, the change in pH may impact the structural confirmation of the enzyme and, thus, its activity, leading to the altered composition of released peptides [28].

Our current study showed how %DH increased with an elevation in incubation temperature and time (Table 2). These results concurred with the results obtained from our previous study [21]. Also, in the current study, papain demonstrated similar proteolytic activity to actinidin, while bromelain appeared to be vastly different. For example, whey protein hydrolysates obtained using bromelain reached only around 9 %DH at 60 °C, whereas papain showed double (around 18 %DH) after 5 h of incubation (Table 2). This clearly explains the difference in the specificity of these proteases towards these substrates despite being categorised in the same CA family group. So, papain has the potential to be used in certain applications where a high DH is required to modify the protein structure to achieve, e.g., hypoallergenicity or functionality. Al-Shamsi, Mudgil, Hassan, and Maqsood [29] also showed that the maximum %DH of camel milk protein hydrolysates obtained using papain (∼ 40%) was almost double that achieved by bromelain (∼ 24%) after 6 h under the same processing conditions.

Papain has Cys_25_ and His_159_ present in its active site, and it preferentially cleaves Ala, Ile, Trp, Phe, Val, Leu and Tyr amino acid residues with the peptide bonds of hydrophobic regions. Papain exhibits a preference for an amino acid with a large hydrophobic side chain at the P2 position; however, it does not accept Val at P1’s position [30]. In the case of actinidin, Cys_25_ and His_162_ are two residues present at the active site behind the cleft in the middle of the domains, and they bind with the substrate to create an enzyme–substrate complex. Actinidin cleaves Phe, Val and Leu. P1’s position is a specificity determinant, and, unlike papain, this enzyme can accept Val at P1’s position [31,32]. However, in the case of bromelain, it has limited substrate specificity, and it only cleaves the carbonyl end of Lys, Ala, Tyr and Gly [32]. 

For MPC, β-CN has a hydrophobic region at the C-terminus of 136-209 residues [33,34]. This sequence contains all three Leu (10), Val (8) and Phe (3) target amino acid residues that are essential for actinidin to act on. For papain, including all three amino acids, the other four amino acids, Ala (2), Ile (3), Trp (1) and Tyr (2), are also present as potential cleavage sites. On the other hand, in the case of bromelain, a very limited number of amino acids (two residues of each Lys, Ala and Tyr and one Gly) is available. For this reason, a greater possibility exists for papain and actinidin to cleave these proteins, resulting in a reduction in the molecular weight of proteins/peptides and an increased %DH, which is also reflected in the results (Table 2). Similarly, in the case of αs_1_-CN, the amino acid fraction from 100 to 199 is considered a highly hydrophobic region present at the carboxyl-terminal [33], which consists of Leu (6), Val (3) and Phe (4) as potential target sites for actinidin and papain. For papain, the other four amino acids, including Ala (5), Ile (5), Trp (2) and Tyr (8), are also present in the primary structure of αs_1_-CN as possible targets. Also, in the same protein fraction, Ala (5) and Tyr (8), Lys (6) and Gly (5) are present and are potential cleavage sites for bromelain to act on. Furthermore, in the case of αs_2_-CN, the amino acid chain of 126 to 207 is considered a highly hydrophobic region [33]. This region contains Leu (5), Val (5), Phe (3) (potential cleavage sites of actinidin and papain) and Ala (2), Ile (3), Trp (1) and Tyr (4) (more cleavage sites for papain to bind with). Whereas in the case of bromelain, despite the presence of adequate amounts of Lys (14), there is still an absence of Gly and with only limited amounts of Ala (2) and Tyr (4) [32], which could contribute to lower proteolysis compared to papain and actinidin (Table 2), as reflected in Table 3. 

When it comes to major whey proteins, β-LG contains two disulphide bridges located at Cys_106_-Cys_119_ and Cys_66_-Cys_160_ that contribute to the stability of this protein [35,36]; however, the cleavage of these disulfide bonds by these proteases can lead to an enhanced susceptibility to hydrolysis [37]. Therefore, actinidin and papain (due to Cys present at their active sites) could have cleaved these disulphide bonds, resulting in the disappearance of bands in the PAGE gels (Table 3) and an overall increment in %DH (Table 2). On the other hand, the cleavage of disulphide bonds is not supported by bromelain [32], and the SDS PAGE of the current study also resulted in the presence of a higher β-LG-remaining protein fraction compared to papain and actinidin (Table 3). α-LA, as proportionally the second most abundant whey protein, is characterised by the presence and abundance of Leu (14), Val (6) or Phe (4) [38], which allows for the greater binding of papain and actinidin with more random action than bromelain, which may contribute towards greater proteolysis (Table 3). The remaining protein representing α-LA in Table 3 disappeared at a greater rate than those of β-LG, which indicated a greater affinity towards α-LA than β-LG. 

Limited proteolysis results in hydrolysates with a low level of DH and is frequently desirable in these cases when improvements of a specific functionality are needed, such as improved emulsifying properties or foam stability. Such modifications assist with the structuring of the final products, such as processed meats, edible films, ice creams, froth drinks and salad dressings [39]. Bromelain, despite showing a very low %DH under the current processing conditions (Table 2), can still be used in applications where small but targeted hydrolysis may be needed, such as an improvement in certain functional properties. For example, salad dressing prepared using 1 %DH whey protein hydrolysates resulted in a creamy texture in the final product compared to the control, which was prepared using unhydrolysed WPC and where the final product appeared very runny with a thin texture. A further slight increase in %DH (remaining between 2–4%) resulted in dressings with desirable stability [40].

It is well known that hydrolysis can proceed at a greater rate if the activation energy is low [41]. The activation energy of actinidin (at both substrate ratios varied between 14 and 17) and papain (∼11–12) for whey proteins appeared substantially lower than those calculated for bromelain (between 23–24) (Table 4). This also supports our results by indicating the range of enzyme efficiency in dairy systems in the following order: papain > actinidin > bromelain.

Also, kinetic parameters (Table 5) are in agreement with the results obtained for %DH, where all three proteases showed a greater cleavage of the peptide bonds in the case of WPC than MPC. Bromelain showed the lowest cleavage action in the case of both substrates compared to actinidin and papain (Table 2). Proteases such as aspartic [42,43] and serine [25] with various substrates obey the Michaelis–Menten kinetics. Only these enzymes show this behaviour and have the ability to bind with a specific substrate due to their specific active sites rather than allosteric sites [25]. A study by Salami et al. [44] showed that a serine protease also showed Michaelis–Menten behaviour during the hydrolysis of milk proteins, including WPC and caseins. The KM values for casein and WPs with chymotrypsin were 0.81 and 3.38 mg mL^−1^, respectively, and with trypsin, 3.78 and 1.37 mg mL^−1^, respectively. In comparison to the current study (Table 5), the addition of actinidin and papain to WPC and MPC suspensions resulted in KM of 2.13 × 10^4^ to 3.18 × 10^4^ and 1.87 × 10^4^ to 3.05 × 10^4^ (Table 5), which is equivalent to 21.3 to 31.8 and 18.7 to 30.5 mg mL^−1^, respectively. However, in the case of bromelain, KM values were about 5 and 6 times greater (WPC and MPC, respectively) than papain and actinidin, which showed their lowest binding affinity compared to the other two proteases. The specificity and activity of an enzyme depends on the substrate and mode of action despite, for example, the fact that these three enzymes belong to the same family and share similar structure and functions [45]. The difference in kcat/KM values amongst all the enzymes suggests that there is a difference between these three enzymes in the strength of substrate binding sites. These *Kcat* differences can contribute to the binding strength [1]. Therefore, the weaker binding of substrates in the productive mode towards papain can result in lower kcat despite having the lowest KM. Kinetic and thermodynamic studies are important for the greater control of reaction rates and are a clearly understood reaction mechanism to optimise the hydrolysis process [25]. Therefore, the optimisation of processing conditions by understanding changes in milk protein conformations and evaluating kinetic and thermodynamic parameters can lead to knowledge of the more appropriate selection of a particular enzyme to achieve beneficial properties, such as changes in functional properties, digestibility or alterations in allergenicity [2]. An example of this is if industries are after obtaining a final product at a faster rate, but thermal stability is not required such as certain products manufactured at lower temperatures. In this situation, kinetics in terms of a lower KM is an important parameter to choose instead of activation energy. However, where a reaction at a faster rate is required at a certain range of temperatures, both thermodynamic and kinetic data provide useful information for the selection of protease/s.

## 5. Conclusions

As this study shows, these three proteases did not express the same proteolytic behaviour toward milk proteins. Papain showed the greatest %DH with whey proteins (∼18% DH), whereas bromelain showed the lowest (∼7%DH) with MPC at 60 °C after 5 h of incubation. The decline in pH was temperature-dependent, with the highest difference of around 0.35 obtained during the hydrolysis of MPC with papain and 0.44 during WPI hydrolysis with bromelain at 60 °C. The SDS-PAGE results revealed α_s_-casein and α-LA were preferential substrate fractions in the case of MPC and whey, respectively. Kinetic and thermodynamic parameters revealed that papain and actinidin behaved similarly and showed greater substrate specificity compared to bromelain. Papain showed the lowest KM, whereas bromelain showed the highest KM value. The kcat/KM values followed an actinidin > papain > bromelain order. WPC was a preferential substrate for all three proteases. Also, the activation energy of papain was lowest, and bromelain was highest. These results indicate that actinidin and papain exert appreciable activities toward dairy proteins and have the ability to be used in certain products where specific characteristics are required. However, further knowledge, such as the sequencing of released peptides, can surely add light to evaluate the behaviour of these enzymes toward a specific substrate.

## Figures and Tables

**Table 1:** The pH decline (final–initial) after 5 h of incubation of suspensions (5% *w*/*w*) of MPC, WPC or WPI with papain or bromelain at an enzyme-to-substrate ratio of 5.21 units g^−1^ and a temperature of 15, 40 or 60 °C.

Temp (°C)	Papain			Bromelain		
	MPC	WPC	WPI	MPC	WPC	WPI
15	0.02 ^aA^	0.02 ^aA^	0.08 ^aB^	0.12 ^aC^	0.12 ^aC^	0.13 ^aC^
40	0.19 ^bBC^	0.17 ^bB^	0.18 ^bB^	0.14 ^abA^	0.15 ^abA^	0.23 ^bD^
60	0.35 ^cE^	0.26 ^cC^	0.30 ^cD^	0.17 ^bcA^	0.21 ^cB^	0.44 ^cF^
SEM	0.01					

SEM—pooled standard error of the mean; upper-case and lower-case superscript letters display significant difference (*p* < 0.05) within a row and a column, respectively.

**Table 2:** Degree of hydrolysis (%DH) reached after 5 h of incubation of (5% *w*/*w*) with suspensions of MPC, WPC or WPI with papain or bromelain at an enzyme-to-substrate ratio of 5.21 units g^−1^ and a temperature of 15, 40 or 60 °C.

Degree of Hydrolysis (%)				
Enzyme	Temperature (°C)	Substrate		
		MPC	WPC	WPI
Bromelain	15	1.67 ^fC^	2.89 ^fB^	3.45 ^fA^
	40	3.82 ^dC^	6.42 ^eB^	7.12 ^eA^
	60	6.45 ^bC^	8.96 ^cB^	9.23 ^cA^
Papain	15	3.23 ^eC^	6.9 ^dB^	8.0 ^dA^
	40	5.38 ^cC^	11.53 ^bB^	12.57 ^bA^
	60	8.12 ^aC^	16.77 ^aB^	17.69 ^aA^
SEM		0.03		

Values are the means of at least 3 independent observations (n ≥ 3); SEM = standard error of the mean. The means in the same column displayed by different capital letter superscripts are significantly different (*p* < 0.05). The means in the same row displayed by different small letter superscripts are significantly different (*p* < 0.05).

**Table 3:** Proportion of milk proteins (%) remaining after hydrolysis of MPC (5% *w*/*w*) or WPC (5% *w*/*w*) with actinidin, bromelain or papain at 60 °C for 5 h. The proportion expressed is relative to the un-hydrolysed control estimated from the reduced SDS-PAGE gels.

Protein	Proportion of Proteins Remaining Relative to Control (%)								
	Temperature (°C)								
	15	40	60	15	40	60	15	40	60
MPC	Proteases								
	Actinidin			Bromelain			Papain		
α_s_-CN	86.0 ^bB^	31.0 ^fD^	16.6 ^hE^	88.7 ^aB^	85.7 ^cA^	72.5 ^dA^	65.3 ^eC^	17.7 ^gD^	9.3 ^iC^
β-CN	87.4 ^aA^	28.4 ^fE^	22.1 ^gB^	70.5 ^bG^	66.2 ^cE^	64.3 ^dC^	49.0 ^eF^	15.5 ^hF^	10.2 ^iB^
κ-CN	75.9 ^bE^	25.3 ^fG^	17.8 ^gC^	78.5 ^aD^	72.0 ^cD^	66.5 ^dB^	62.7 ^eD^	10.5 ^hG^	10.2 ^iB^
β-LG	83.5 ^aC^	40.4 ^fB^	25.1 ^hA^	81.8 ^bC^	75.5 ^dC^	72.4 ^eA^	78.0 ^cA^	26.7 ^gB^	19.6 ^iA^
α-LA	80.6 ^aD^	45.5 ^fA^	17.0 ^hD^	77.3 ^bE^	76.8 ^cB^	57.0 ^eD^	68.5 ^dB^	32.7 ^gA^	10.2 ^iB^
**WPC**	**Actinidin**			**Bromelain**			**Papain**		
β-LG	48.2 ^bF^	34.2 ^eC^	16.2^hF^	76.8 ^aF^	42.0 ^cF^	37.7 ^dE^	28.4 ^fG^	19.3 ^gC^	8.9 ^iD^
α-LA	47.8 ^cG^	26.3 ^fF^	6.1^hG^	94.1 ^aA^	37.6 ^dG^	35.5 ^eF^	54.0 ^bE^	17.2 ^gE^	5.3 ^iE^

The standard error of the mean (SEM) of at least three independent observations for hydrolysates of MPC and WPC was 0.191 and 0.192, respectively; lower- and upper-case superscript letters indicate significant difference (*p* < 0.05) within a row and a column, respectively.

**Table 4:** Reaction constants (*k*), coefficient of correlation (r^2^), Arrhenius activation energies (E_a_) and pre-exponential factors (A) established from the Arrhenius plots for the hydrolysis of proteins in 5% (*w*/*w*) suspensions of MPC, WPC or WPI with actinidin, bromelain or papain at an enzyme-to-substrate ratio of 5.21 units g^−1^ of protein and a temperature range between 15 and 60 °C.

Enzyme	Substrate	Temp °C	*K*	r² ^(1)^	Ea	A	r² ^(2)^
			(×10^−6^, s^−1^)		(kJ mol^−1^)		
**Actinidin (5.21 Units g^−1^ of protein)**	**MPC**	15	3.46	0.97	18.09	0.0079	0.81
		35	8.66	0.97			
		40	9.14	0.94			
		55	9.71	0.97			
		60	9.99	0.93			
	**WPC**	15	6.98	0.94	15.02	0.0037	0.99
		35	10.07	0.9			
		40	11.62	0.91			
		55	15.37	0.89			
		60	15.77	0.9			
	**WPI**	15	6.78	0.89	14.38	0.0028	0.98
		35	10.54	0.9			
		40	11.84	0.9			
		55	14.49	0.89			
		60	15.15	0.89			
**Bromelain (5.21 Units g^−1^ of protein)**	**MPC**	15	0.4	0.96	25.39	0.0158	0.99
		40	0.93	0.97			
		60	1.65	0.92			
	**WPC**	15	0.49	0.81	23.91	0.0112	0.96
		40	1.33	0.99			
		60	1.86	0.94			
	**WPI**	15	0.75	0.78	24.52	0.0009	0.98
		40	1.47	0.98			
		60	1.92	0.96			
**Papain (5.21 Units g^−1^ of protein)**	**MPC**	15	0.63	0.99	13.68	0.0002	0.97
		40	0.9	0.96			
		60	1.38	0.99			
	**WPC**	15	1.43	0.95	12.07	0.0002	0.99
		40	2.04	0.98			
		60	2.83	0.98			
	**WPI**	15	1.65	0.99	11.05	0.0002	0.96
		40	2.16	0.95			
		60	3.11	0.97			

^(1)^ Coefficient of determination for *k*; ^(2)^ Coefficient of determination for Ea_._

**Table 5:** Michaelis–Menten kinetic parameters describing the hydrolysis of the milk protein concentrate (MPC) and whey protein concentrate (WPC) suspensions (5% *w*/*w*) using actinidin, bromelain or papain (2.6 units of enzyme activity g^−1^) with substrate concentrations of 20, 40, 60, 80 or 100 mg mL^−1^ determined at 60 °C.

Enzyme	Substrate	Vmax	1/2 Vmax	KM	Kcat	Kcat/KM
		(μg mL ^−1^ min^−1^)	(μg mL^−1^ min^−1^)	(×10^−4^, μg mL^−1^)	(min^−1^)	(mL min^−1^ μg^−1^)
Actinidin	WPC	3.96	1.98	2.13	0.99	46
	MPC	1.56	0.78	3.18	0.39	12
Bromelain	WPC	1.29	0.65	10.71	0.32	3
	MPC	1.01	0.50	17.71	0.25	1
Papain	WPC	1.12	0.56	1.87	0.28	15
	MPC	1.28	0.64	3.05	0.32	11

## Data Availability

Data is contained within the article and supplementary material.

## Supplementary Materials

The following supporting information can be downloaded at https://www.mdpi.com/article/10.3390/foods12234248/s1. Table S1: Reaction constants (*k*), coefficient of correlation (r^2^), Arrhenius activation energies (E_a_) and pre-exponential factors (A) established from the Arrhenius plots for the hydrolysis of proteins in 5% (*w*/*w*) suspensions of MPC, WPC or WPI with actinidin at an enzyme-to-substrate ratio of 1.04 units g^−1^ of protein and a temperature range between 15 and 60 °C.
