# Supplementary material for: Milk Protein Hydrolysis by Actinidin—Kinetic and Thermodynamic Characterisation and Comparison to Bromelain and Papain
Source: Foods. 2023 Nov 24;12(23):4248. doi: 10.3390/foods12234248 (PMC10706065; doi:10.3390/foods12234248)
Supplement: Supplementary file 1 [file foods-12-04248-s001.zip › foods-2703477-supplementary.pdf]

Table S1: Reaction constants (*k*), coefficient of correlation (r^2), Arrhenius activation energies (Ea) and pre-exponential factors (A) established from the Arrhenius plots for hydrolysis of proteins in 5% (w/w) suspensions of MPC, WPC or WPI with actinidin at an enzyme to substrate ratio of 1.04 Units g^{-1} of protein and a temperature range between 15 and 60°C.

**Enzyme**	**Substrate**	**Temp °C**	***k* ($x10^{-6}$, s^{-1})**	**$r^{2\ (1)}$**	**Ea (kJ mol^{-1})**	**A**	**$r^{2\ (2)}$**
Actinidin (1.04 Units g^{-1} of protein)	MPC	15	3.95	0.95	21.07	0.0273	0.97
		35	8.38	0.92			
		40	9.3	0.96			
		55	11.88	0.94			
		60	13.63	0.98			
	WPC	15	5.37	0.87	17.14	0.0068	0.99
		35	9.2	0.87			
		40	9.19	0.89			
		55	12.93	0.86			
		60	14.18	0.86			
	WPI	15	6.1	0.92	15.28	0.0034	0.98
		35	9.14	0.95			
		40	10.07	0.93			
		55	13.35	0.93			
		60	14.63	0.87			

(1) Coefficient of determination for *k*; (2) Coefficient of determination for Ea
